# The Greater Diseases of the Liver

**Published:** 1891-12

**Authors:** 


The Greater Diseases of the Liver: Jaundice, Gall Stones, Enlargements, Tumors, and Cancer; and their Treatment. By J. Compton Burnett, M. D. Philadelphia: Hahnemann Publishing House, 1891. Price, 60 cents.

This beautiful little volume has been dedicated to the memory of Rademacher, the resuscitator of Paracelsic Organopathy, by the author. Dr. Burnett is indeed a prolific writer, but, unfortunately, not always homœopathic in his expressions. As the book is sold at a low figure, it will pay physicians of any school to buy it. There is much in it that is good. W. S.

